# Challenges to providing quality substance abuse treatment services for American Indian and Alaska native communities: perspectives of staff from 18 treatment centers

**DOI:** 10.1186/1471-244X-14-181

**Published:** 2014-06-17

**Authors:** Rupinder Legha, Ashley Raleigh-Cohn, Alexandra Fickenscher, Douglas Novins

**Affiliations:** 1 Department of Psychiatry, University of Colorado, 13001 E. 17th St., MSF546, Building 500, Aurora, CO 80045, USA; 2 Centers for American Indian and Alaska Native Health, University of Colorado Anschutz Medical Campus, Mail Stop F800, Nighthorse Campbell Native Health Building, 13055 E. 17th Ave., Aurora, CO 80045, USA

**Keywords:** Indians, North American, Substance abuse treatment centers, Health services research, Organizational case studies

## Abstract

**Background:**

Substance abuse continues to exact a significant toll, despite promising advancements in treatment, and American Indian and Alaska Native (AI/AN) communities remain disproportionately impacted. Understanding the challenges to providing quality substance abuse treatment to AI/AN communities could ultimately result in more effective treatment interventions, but no multi-site studies have examined this important issue.

**Methods:**

This qualitative study examined the challenges of providing substance abuse treatment services for American Indian and Alaska Native (AI/AN) communities. We conducted key informant interviews and focus groups at 18 substance abuse treatment programs serving AI/AN communities. Seventy-six service participants (21 individuals in clinical administrative positions and 55 front-line clinicians) participated in the project. Interview transcripts were coded to identify key themes.

**Results:**

We found that the challenges of bringing effective substance abuse treatment to AI/AN communities fell into three broad categories: challenges associated with providing clinical services, those associated with the infrastructure of treatment settings, and those associated with the greater service/treatment system. These sets of challenges interact to form a highly complex set of conditions for the delivery of these services.

**Conclusions:**

Our findings suggest that substance abuse treatment services for AI/AN communities require more integrated, individualized, comprehensive, and longer-term approaches to care. Our three categories of challenges provide a useful framework for eliciting challenges to providing quality substance abuse treatment in other substance abuse treatment settings.

## Background

Despite promising advances in biopsychosocial treatments, substance use disorders continue to significantly impact the health of people worldwide, due in part to the treatment gap between optimal care and currently available services [1]. For example, in the United States, the Institute of Medicine’s 2006 *Improving the Quality of Health Care for Mental and Substance-Use Conditions* concluded that improper dissemination of and critical gaps in the existing evidence base result in ineffective treatment practices [2]. Other issues reducing the quality of substance abuse treatment include discrimination and stigma, poor coordination of substance abuse services with mental health and medical care, inconsistent licensing requirements, and an inadequately trained workforce [2-4]. Unlike the majority of medical care in the United States, substance abuse treatment relies primarily on public rather than private sources, resulting in chronic underfunding. The substance abuse treatment infrastructure is further undermined by high rates of staff turnover and treatment center closures and by overwhelming data collection requirements for accreditation and reimbursement [3,5,6]. Additionally, unlike other developed countries such as the United Kingdom, in the United States no centralized authority mandates the implementation of uniform standards or evidence-based practices [7]. Though substance abuse disorders are chronic conditions often associated with complex medical and psychiatric comorbidities as well as severe impairment in multiple areas of functioning [8,9], the current service model provides inadequate, short-term services that fail to address patients’ long-term addiction treatment and social support needs [10-12]. Furthermore, there are too few programs available to those who need treatment [13].

American Indian and Alaska Native (AI/AN) communities face additional challenges in pursuing quality substance abuse treatment, including high levels of need. AI/ANs suffer disproportionately from substance use disorders and their physical and emotional health consequences [14-16]. Only 1 in 8 AI/AN adults needing treatment receives it at a specialized facility [17], a rate comparable to the national average. However, AI/AN communities’ health services receive significantly lower per capita spending than health services in the rest of the United States, meaning their substantial needs are not matched by a comparable commitment of resources [18,19]. Treatment barriers further interfering with meeting these needs include geographical remoteness, poverty, poor transportation infrastructures, and a shortage of qualified providers. Due to their history of oppression and mistreatment, AI/AN communities also harbor mistrust towards institutional sources of care [20,21]. Many AI/AN people may question the utility of western approaches to healing and instead rely on traditional, indigenous approaches exclusively [20]. Evidence-based practices could potentially improve services, but AI/AN treatment programs and academics have raised concerns regarding their cultural appropriateness, inadequate guidance to adapt them for use with AI/AN patients, and even their applicability to AI/AN populations, which are rarely included in research efforts [22,23].

Understanding the challenges service providers face in their efforts to provide quality substance abuse treatment services to AI/AN communities could ultimately result in the design of more effective care. In a previous analysis of data from this same study, we identified specific challenges to addressing cultural issues in treatment in these communities. These included the within-community diversity of AI/ANs, lack of available resources to provide culturally-based services (e.g., access to traditional healers), and pressure from funders to focus on evidence-based practices [24]. To our knowledge, no multi-site studies have addressed this important issue of challenges to providing quality substance abuse services for AI/AN communities more broadly. This study's purpose is to use qualitative data analyses of 21 interviews and 10 focus groups at 18 treatment centers nationwide to identify the challenges alcohol and substance abuse treatment centers for AI/AN communities face in providing meaninful and effective treatment.

## Methods

Data for these analyses come from the second phase of the Centers for American Indian and Alaska Native Health’s *Evidence-Based Practices and Substance Abuse Treatment for Native Americans* project. This study’s primary aims are to: a) describe the use of specific evidence-based treatments (EBTs) in substance abuse treatment programs serving American Indian and Alaska Native communities; b) describe the factors associated with the implementation of evidence-based treatments in these programs; and c) identify methods for more effective dissemination of evidence-based treatments to substance abuse treatment programs serving American Indian and Alaska Native communities. This project also examines how treatment programs design, implement, and assess their services and incorporate evidence-based concepts and traditional healing techniques into these services [24].

An Advisory Board supports this project. Members include administrators, providers, evaluators from the AI/AN substance abuse treatment community, and researchers with expertise in AI/AN substance abuse treatment and dissemination research. Phase 1 consisted of extensive Advisory Board discussions of the issues related to delivering quality substance abuse treatment services in AI/AN communities and the place of EBTs in these services [22]. Phase 2 consisted of “program case studies” involving visits to treatment programs and qualitative data collection about the communities served, services offered, challenges to delivering these services, and EBT use [24]. In the third and final stage of the project, clinical directors of 445 behavioral health programs nationwide were asked to complete a 45-minute survey about their program and experience with EBTs [25]. Data for these analyses are drawn from the Phase 2 program case studies.

### Settings and participants

For Phase 2, the Advisory Board identified programs based on their reputations for innovative clinical services and to assure adequate representation of the geographic, cultural, and reservation/rural/urban diversity of AI/AN communities. The criteria for innovative clinical services included a) combining cultural and biomedical treatment approaches, b) incorporating evidence-based practices into treatment, and c) receiving competitive federal funding for service implementation and expansion. Our initial letters of inquiry, which were directed to program leadership at eighteen substance abuse treatment programs, explained the study’s purpose, invited programs to participate, and explicitly reassured programs, their host tribes/organizations, and individual participants of their confidentiality [26,27]. All eighteen substance abuse treatment programs invited to participate in the program case study component of this study agreed to participate. We deliberately emphasized confidentiality throughout this study in order to maximize participation and minimize organizational and participant discomfort. Such discomfort is not uncommon in AI/AN communities due to prior research abuses and the stigma of mental illness [21,22]. Accordingly, for the focus group and key informant interviews, we did not collect individual participants’ demographic information, and instead focused on the key topics of interest for this study. Focus groups included program staff in front-line clinical positions. Because of the small size of many programs, we purposely included representatives from multiple treatment programs within the same tribe, tribal consortium, or urban organization in a single focus group. Though this approach required more effort from the interviewer to collect specific information about each program, we prioritized having a larger group to facilitate richer conversation over much smaller program-specific focus groups. At each of the eighteen participating programs, we also conducted one or more key informant interviews with program staff in clinical administrative positions. We chose to focus on interviews of staff members and did not interview patients receiving services because of this study’s primary aim of describing the use of specific evidence-based treatments in these substance abuse treatment programs. Furthermore, evaluating staff perspectives represents a practical starting point for understanding the challenges of providing quality substance abuse treatment. Data collection took place from August 2009 through July 2010.

As indicated in Table 1, these eighteen programs reside within seven out of the twelve Indian Health Service (IHS) regions. These twelve IHS regions are described in further detail on the IHS website (http://www.ihs.gov/images/cultureofcaring.jpg). To maintain program confidentiality, we identify each IHS region as IHS Region A, B, C, etc. Twelve were outpatient programs and six provided residential treatment. Tribes or tribal non-profit organizations operate these programs, which all receive additional funding from IHS. Ten programs are located on reservations, 3 in non-reservation rural areas, and 5 in urban areas. Participants reported that their programs had as many as 19 and as few as 3 staff and as many as 200 and as few as 12 clients. Twenty-one individuals in clinical administrative positions participated in key informant interviews, and 55 front-line clinicians participated in 10 focus groups (a total of 76 participants).

**Table 1:** Characteristics of the programs, key informants, and focus group interviews

**IHS funding region**	**Number of outpatient programs**	**Number of residential programs**	**Number of key informants interviewed**	**Number of focus group interviews**	**Total number of people in focus groups**
**A**	1	1	5	1	4
**B**	2	1	4	2	9
**C**	1	1	5	1	7
**D**	5	1	1	1	13
**E**	1	0	1	1	9
**F**	1	2	4	3	10
**G**	1	0	1	1	3
**Totals**	12	6	21	10	55

### Ethical considerations

The Colorado Multiple Institutional Review Board reviewed and approved all procedures, which also underwent local review processes that always included an administrative review and sometimes included review by a research review committee or a formal institutional review board. After reviewing a complete description of the study, each participant completed written informed consent.

### Data collection

The Advisory Board developed the Phase 2 Focus Group and Key Informant Interview Guides to generate open-ended conversations about the community the program serves, the services provided and how they were developed, the challenges of providing these services, and the participants' experience with selected EBTs. These guides can be accessed on the project's web page: http://www.ucdenver.edu/academics/colleges/PublicHealth/research/centers/CAIANH/EBP/Pages/ProjectMeasures.aspx. Examples of EBTs queried include 12-Step Facilitation [28], Cognitive Behavioral Therapy [29], Matrix Model [30], and Motivational Interviewing [31]. The interview and focus group guides included open-ended “stem” questions designed to steer the conversation through key topics of interest to the research team and “probes” to explore specific issues not spontaneously identified by participants (e.g., whether the program felt pressure to provide specific treatments because of accreditation or reimbursement requirements). Key informant interviews lasted 60 minutes and focus groups lasted 90 minutes. Project directors had the option of distributing interview/focus group guides prior to the research team’s visit. The principal investigator, a psychiatrist who has worked with AI/AN communities for over fifteen years, conducted all interviews, which were recorded. Detailed notes were taken as a backup. For their participation, programs received clinical and/or training materials worth up to US $300.

### Data analyses

All interviews were recorded and transcribed. In only one instance was the recording quality so poor that we relied on detailed notes for analytic purposes. After transcription all recordings were destroyed. The authors analyzed the transcripts using NVivo [32]. Utilizing the principles of Grounded Theory [33], interview transcripts were coded for key themes relevant to the purpose of this study [34-37]. Initial codes were broken down into smaller categories and used to inform emerging hypotheses. Regular discussions among the authors were held to achieve consensus on emerging themes and hypotheses. Additional file 1 demonstrates how this paper adheres to RATS qualitative research guidelines, which are available online at http://www.biomedcentral.com/authors/rats.

## Results

We identified three sets of challenges for bringing effective substance abuse treatment to AI/AN communities: challenges associated with providing clinical support, challenges associated with the infrastructure of the treatment settings, and challenges associated with the service/treatment system. These are summarized in Table 2. Of particular importance was the way these different sets of challenges interact synergistically with one another, creating a highly complex context for the delivery of these services. We first present these three sets of challenges separately for clarity and organization and then discuss their interrelatedness.

**Table 2:** Themes, subthemes, and examples: challenges to providing quality substance abuse treatment to AI/AN communities

**Themes**	**Subthemes**	**Examples**
Challenges associated with providing clinical support	Barriers to treatment seeking and engagement: sociodemographic	Homelessness
		Lack of transportation
		Legal issues
		High relapse
		Unemployment
	Barriers to treatment engagement and process	Complex trauma histories
		Diversity of patients
		Integrating culture into services
		Lack of motivation
		Stigma of treatment
Challenges associated with the infrastructure of treatment settings	Frontline worker challenges: fatigue and burnout	Emotional and personal investment
		High caseloads
		Paperwork and administrative responsibilities
		Professional boundaries
		Shortage of staff
	Lack of program and treatment resources	Inadequate length of treatment
		Lack of office supplies and space
Challenges associated with the service/treatment system	Barriers associated with providing	Limited housing options
	Adequate aftercare	Limited transportation resources
		Limited treatment options
	Appropriateness of treatments	Pressure to use Evidence-Based Treatments (EBTs)
	Excessive and unconstructive paperwork	Interference with clinical care
		Not pragmatic or useful

### Challenges associated with providing clinical support

#### ***Barriers to treatment seeking and engagement: socio-demographic***

Participants cited AI/AN communities’ socioeconomic challenges as a primary obstacle to pursuing treatment. “There are so many survival needs that come first—housing, a job, food,” one provider explained, adding, “Things like outpatient treatment are probably last on the list” (Region E, Focus Group 1). Reiterating this idea, another provider observed, “People just do not have cars, gas, money, the ability to come here when they’re managing tough situations at home, kids, getting here” (Region E, Focus Group 1). As a result, providers indicated, only a small percentage of people in need of treatment actually receive it; and among the few who do receive it, treatment is often undermined. Capturing clients’ predicaments, one provider explained, “If I don’t have a roof over my head, then I don’t really care about finding my inner being, you know what I mean?” (Region F, Key Informant 3). Criminal records prevent clients from receiving employment, housing, and drivers’ licenses. These housing, transportation, and employment deficits, in turn, hinder a solid community-level foundation for treatment and sobriety. “Sometimes I forget they’re [h]ere for treatment. I’m just trying to get them a job or . . . to finish their GED . . . because [once they] get out of treatment, if they don’t have any of those things, they’re going to relapse most likely” (Region C, Key Informant 5). Because relapse rates and prevalence of misuse remain high, and substance abuse, considered a community-wide problem, persists: “You get one off the street and there’s two more to take his or her place” (Region C, Key Informant 5).

#### ***Barriers to treatment engagement and process***

Individual trauma histories coupled with AI/AN communities’ shared history of oppression result in complex mental health and substance abuse problems that impact the treatment process:

“I grew up in an alcoholic home, I was raised in a foster home, I was in a boarding school, I may have had sexual abuse, I may have been physically abused or emotionally abused . . . .” That’s what’s walking in your door. It’s not simple. It’s not “I’m drinking a six-pack a day and I really get drunk on the weekends. Help me sober up.” [W]hat’s coming to light for our communities is the trauma that has happened for so many generations. . . . [S]o how do we fix that? (Region D, Key Informant 1)

Numerous providers cited clients’ “trauma upon trauma upon trauma” (Region B, Focus Group 1) and how these complex trauma histories can result in a significant mistrust of providers as well as additional mental health treatment needs.

Court-ordered treatment, mandated for a substantial percentage of clients, was described by study participants as undermining motivation, while stigma towards mental health and substance abuse treatment further precludes pursuing and continuing in treatment. Providers noted the additional challenge of bringing culture into services, due to the tremendous cultural and geographical diversity of AI/AN communities, and the fallacy of perceiving “all Indians as being Indians, rather than [understanding] we have 500 tribes in the United States” (Region F, Focus Group 1). Integrating culture, critical for the many AI/AN clients who prefer traditional rather than western approaches to treatment, is also hindered by limited cultural resources. Providers cited few local traditional healers and limited access to sweat lodges and powwows, most often due to transportation and funding deficits.

### Challenges associated with the infrastructure of the treatment settings

#### ***Frontline worker challenges: fatigue and burn out***

Serving clients with substantial, interrelated socioeconomic, substance abuse, and mental health needs demands considerable emotional investment from staff. As one provider elaborated, “you have to have a certain amount of flexibility and willingness to wade through the mud and muck of peoples’ lives every day because these people come in here when everything is falling apart” (Region B, Key Informant 4). Providers explained that personal commitment to clients’ treatment requires additional responsibility to maintain professional boundaries. It can also cause caregiver fatigue, resulting in high rates of turnover. A shortage of qualified staff, transportation barriers, and insufficient salaries yield chronic staff openings. As a result, “3 people have to do the work of 5” (Region C, Focus Group 1), thereby compounding caregiver fatigue.

Overwhelming paperwork and administrative demands, consuming up to 50% of time, further interfere with treatment, leaving providers feeling like “we’re constantly running against the clock,” “fighting to keep [our] nostrils above the proverbial water line” (Region C, Key Informant 4). The combination of high caseloads, patients with substantial needs, and significant administrative duties results in staff burnout related to feelings of ineffectiveness. Regarding her inability to meet clients’ needs, one provider commented, “I feel the desperation of people’s voices on the other end of the phone when they say they have to get intro treatment now and I have to tell them three to five months. . . . I hear their hearts drop on the floor” (Region C, Key Informant 4). Several program directors cited the need to provide staff with “mental health days” to recuperate and additional praise and support to sustain their efforts.

#### ***Lack of program and treatment resources***

Limitations in programs’ physical infrastructure and other treatment resources add to workplace demands. Inadequate or poorly configured physical space (office space, waiting rooms, dining rooms, and group rooms) interferes with client confidentiality and programming. Computers, electronics, and kitchen supplies are often lacking, and training and treatment planning opportunities for clinical staff are limited. Capturing a common sentiment, one program director explained, “I’m always looking for funding. . . . It’s a huge challenge trying to provide everything you need….” (Region F, Key Informant 3), such as revamping treatment curricula or integrating novel treatment approaches. Specific limitations on the length of treatment (e.g., number of days in residential treatment, number of sessions in outpatient treatment) interfere with meeting clients’ significant vocational, housing, and treatment needs. “It’s a challenge to set treatment goals and to try to achieve them in 28 days, one provider noted, “because just about the time you get to know them and see a little progress, they’re ready to go” (Region F, Focus Group 1).

### Challenges associated with the service/treatment system

#### ***Barriers associated with providing adequate aftercare***

Numerous providers cited difficulties with aftercare, specifically limited housing and treatment options. Because housing is considered critical for sober living, inadequate housing resources impact lengths of stay:

[Clients] know if they go back to their homeland there’s all the drinking and drug use going on [so they] relocate, [but] sometimes we have people staying three weeks to a month later waiting for housing because of the [lack of] availability and the funding. (Region A, Focus Group 1)

Limited treatment options exacerbate transitions between residential and intensive outpatient or community reintegration, as well as between detoxification and treatment. They also result in lengthy waitlists and unmet treatment needs, particularly for pharmacologic treatment. “We’re lacking beds and treatment slots,” one provider explained, adding, “More and more people are saying ‘I need help,’ but that help isn’t there . . . because our waitlists are tremendous” (Region C, Key Informant 4). Transportation problems, including vast distances between the few treatment facilities and clients’ limited transportation resources, represent an additional barrier. Providers noted that some clients travel several hours each way, sometimes by foot or bicycle, to receive care.

#### ***Appropriateness of treatments***

Funding sources frequently require using evidence-based treatments (EBTs), but numerous programs expressed concern about their applicability to AI/AN programs and their lack of flexibility, considered critical for working with AI/AN communities’ diverse needs. They also noted how EBTs have not been studied in AI/AN communities. “It’s all good and well to have evidence-based treatment,” one provider explained, “but for who? Who does it work for? . . . You’ve got to realize that it’s different in each community” (Region G, Focus Group 1). The pressure to use one EBT exclusively also contradicts providers’ tendency to, instead, “take a little from everything—from Matrix [an evidence-based program for substance abuse [30]], from Red Road [an AI/AN adaptation of 12-step treatment approaches [38,39]], from whatever you can find” (Region D, Focus Group 1) in order to individualize treatment. Funding requirements to use EBTs combined with concerns about their applicability lead providers to feel “pushed into a corner” (Region F, Focus Group 3) and additionally burdened by treatment requirements that do not fit clients’ needs. “It may not be evidence-based,” one provider explained, “but what we’re doing works. . . . I don’t know how to make it evidence-based [but] if an expert came in who could figure out how to do [that], that would be great” (Region E, Key Informant 1).

#### ***Excessive and unconstructive paperwork***

Administrative demands from government and funding agencies also hinder the personal connection deemed critical for collaborating with clients and facilitating treatment:

We’re burdened with the paperwork, a treatment plan, and everything being done in a timely manner, especially since we’re billing the State Mental Health Service . . . . [S]o they came and look at our files and all of that and they want these things done so we’re under pressure to do that. So we’re trying to balance that out with what the person really needs and how to connect with them. (Region F, Focus Group 2)

In addition to detracting from personalized and individualized care, funding agencies’ outcome measures also fail to portray the progress made. “What’s on paper does not show what happens in the lobbies and hallways” (Region C, Key Informant 4) one provider explained. Another provider noted, “sometimes the outcomes are . . . like a baby being born drug free . . . and that’s not something that you can necessarily measure by a survey” (Region F, Key Informant 3). Capturing the clash between clinical responsibilities and administrative burdens, another provider emphasized, “You don’t provide a service just because it helps your numbers. [You] provide a service because you have the heart to help an individual get better” (Region C, Key Informant 4).

### Complex and interrelated challenges: a summary

Participants’ descriptions underscored significant clinical, program infrastructural, and service system challenges that interfere with their efforts to provide quality individualized and personalized treatment. Limited availability of housing, employment opportunities, and transportation (challenges associated with the service system/treatment system) interfere with treatment and hinder the necessary community-level foundation for sobriety, while complex trauma histories and diverse cultural needs (challenges associated with providing clinical support) create additional demands on programs that are underfunded and overextended. These demands also impact the work force, which struggles with high case loads and excessive administrative responsibilities and thus lacks the time, resources, and emotional reserve to provide quality care (challenges associated with the infrastructure of treatment settings). The pressure to implement EBTs and to monitor outcomes, which creates additional demands on clinicians and programs and which participants perceived as being of questionable clinical significance (challenges associated with the service/treatment system), further undermine care, which is itself often time-limited and difficult to access. As a result, patients are left with unmet needs, and providers feel ineffective.

The following quotations further illustrate how clinical, program infrastructure, and service system challenges interact with one another:

The caseloads are too big. Everything is rush[ed] [and] the quality is not there…. And yet, [funding and regulatory agencies] expect that [providers] know the evidence-based practices, they facilitate and administrate them, and that they do the quality one-on-one care, and the case management and the referral on top of regular case staffings, discharge summaries, phone calls to the community, and helping people pick up the pieces of their lives. [Providers] can’t do that with 25 people. . . . [Agencies] need to let us slow down and do more quality work with people’s lives…. I think that’s why we have the recidivism that we do. We’re not . . . giving [clients] what they need because our case managers are overworked. . . . Our people are traumatized, . . . hugely traumatized. . . . What they need is a lot of TLC coming in. (Region C, Key Informant 5).

This quotation illustrates how high caseloads and excessive clinical responsibilities (challenges associated with the infrastructure of treatment settings) combined with the pressure to use EBTs (challenges associated with the service/treatment system) undermine the provision of quality substance abuse treatment critical for patients with complex trauma histories and high recidivism rates (challenges associated with providing clinical support).

[M]ost of our clients have [substance-abused related] felonies [and they] can’t . . . qualify for public housing. [Clients] get clean, [try] to get [custody of] their kids [and try] to work full time, but there is not suitable housing for them that they can afford. . . . [A] lot of their family members or past friends may have been users [and that’s] counterproductive for them to go and live with a past user. . . . [B]ut they still have to have a roof over their head. . . . [It] can be real frustrating, very frustrating. And it’s frustrating on our part too because we’re looked at as “well, we’re coming to you for help,” but the resources just aren’t there. . . . [W]e don’t have a resource to help with that. (IHS Region F, Key Informant 3)

As illustrated in this quotation, the absence of housing resources (challenges associated with the service/treatment system) prevents clients with complex socioeconomic challenges from leaving communities with endemic substance abuse problems (challenges associated with providing clinical support), consequently thwarting their attempts at sobriety. As a result, providers are left feeling frustrated and ineffective (challenges associated with the infrastructure of treatment settings), while clients' considerable needs remain unaddressed.

## Discussion

Our most striking finding is the powerful, synergistic interactions between clinical, infrastructural, and service system challenges, which create a highly complex context for the provision of quality substance abuse treatment. These challenges’ interrelated nature underscores the need for integrated, comprehensive, and longer-term treatment that address the chronic, relapsing nature of substance use disorders and their complex medical, psychiatric, and social comorbidities [11,40,41]. For example, integrated care models can combine medical, psychiatric, and substance abuse treatment to enhance coordination of care [42], while telepsychiatry can provide culturally appropriate services that traverse transportation and other socioeconomic barriers [43-45]. Community outreach and engagement efforts can help minority communities overcome stigma and mistrust and develop effective, culturally relevant treatment interventions [46,47].

Clinical factors related to cultural differences and trauma histories [48,49]; socioeconomic factors, such as transportation and housing [50-52]; and policy measures [7] have been identified individually as important barriers to care in diverse settings. However, our findings suggest that these challenges are best understood when we are mindful of their interrelatedness with one another. This approach may be particularly important for addressing minority and other underserved communities disproportionately afflicted by substance abuse, where economic, social, and racial realities underlie disparities in care [53,54].

Several of the challenges we identified have been identified by others as ones that are likely unique to AI/AN communities. These include the tremendous need for substance abuse services, mistrust of providers and stigma towards substance abuse treatment, limited funding and other resources, and questions regarding the applicability of evidence-based practices [15,20-23]. We believe several other challenges not previously noted--complex trauma histories, AI/AN communities’ diversity, integrating culture into treatment, and the pressure to use EBTs (appropriateness of treatments)--may also be unique to providing substance abuse treatment to AI/AN communities. However, the majority of the challenges identified likely apply to the general substance abuse patient population in the United States. Such challenges that have been previously cited include clients’ extensive socioeconomic needs [10-12] and treatment programs’ insufficient (qualified) staff and inadequate resources [2-4]. Service/treatment systems’ limited aftercare options, inadequate services to address medical and psychiatric comorbidities [2,13], overwhelming paperwork demands, [6] and poor integration between all phases of care from detoxification to community reintegration have also been noted. Because we did not include programs serving non-AI/AN communities in this sample, this partitioning of factors into general and AI/AN-specific categories is largely dependent upon the admittedly limited literature in this area, and some of the factors that we identify as AI/AN-specific may in fact apply to substance abuse services for other populations, particularly those focused on serving other indigenous communities.

Additional study limitations that should be noted include our focus on programs providing innovative services, which may limit the applicability of these findings to programs that do not meet this criterion. Quantitative data from Phase 3 of this project will be better able to describe the full scope of these issues, though without the specific examples and detailed contexts provided by the qualitative analyses presented here. In addition, we did not interview clients or community members, who would add an important perspective on substance abuse treatment services. This was consistent with this project’s primary goal of describing the use of evidence-based treatments in these programs, however the inclusion of such interviews should be seriously considered in future research.

## Conclusions

This first multisite study of substance abuse treatment programs serving AI/AN communities provides important insights into the considerable challenges these programs face in providing quality care. Challenges associated with providing clinical support, those associated with the infrastructure of treatment settings, and those associated with the service/treatment system interact to form a highly complex set of conditions for the delivery of these services. Our findings suggest that substance abuse treatment services for AI/AN communities require more integrated, individualized, comprehensive, and longer-term approaches to care. Our framework for conceptualizing these challenges may be useful for exploring these issues in other diverse substance abuse treatment settings, enabling clinicians, treatment programs, and policymakers to better assure the provision of the highest quality substance abuse treatment services.

## Competing interests

This work was supported in part by grant R01-DA022239 from the National Institute on Drug Abuse (Dr. Novins). Funds from the American Psychiatric Association/Substance Abuse Mental Health Services Administration Minority Fellowship (Dr. Legha) subsidized the article-processing charge for this paper.

## Authors’ contributions

RL participated in the literature review, data analyses, and writing of this paper. AR participated in the literature review and data analyses. AF participated in the design, data collection, and data analyses for this project, DN is the Principal Investigator on the grant and oversaw all research activities, facilitated all focus groups and key informant interviews, analyzed the qualitative data, contributed to the writing of the manuscript, and read all drafts. All authors read and approved the final manuscript.

## Pre-publication history

The pre-publication history for this paper can be accessed here:

http://www.biomedcentral.com/1471-244X/14/181/prepub

## Supplementary Material

Additional file 1 Adherence to RATS Guidelines, “Challenges to Providing Quality Substance Abuse Treatment Services for American Indian and Alaska Native Communities: Perspectives of Staff from 18 Treatment Centers”. Click here for file
