# Supplementary material for: Challenges to providing quality substance abuse treatment services for American Indian and Alaska native communities: perspectives of staff from 18 treatment centers
Source: BMC Psychiatry. 2014 Jun 17;14:181. doi: 10.1186/1471-244X-14-181 (PMC4080609; doi:10.1186/1471-244X-14-181)
Supplement: Additional file 1 — Adherence to RATS Guidelines, “Challenges to Providing Quality Substance Abuse Treatment Services for American Indian and Alaska Native Communities: Perspectives of Staff from 18 Treatment Centers”. [file 1471-244X-14-181-S1.docx]

RATS GUIDELINES, "Challenges to Providing Quality Substance Abuse Treatment Services for American Indian and Alaska Native Communities: Perspectives of Staff from 18 Treatment Centers," Legha et al. (Please note, the line numbers refer to the document "Challenges BMC Version, Revisions.doc"

| **ASK THIS IN THE MANUSCRIPT** | **THIS SHOULD BE INCLUDED IN THE MANUSCRIPT** | **AUTHOR'S RESPONSE** | |
| --- | --- | --- | --- |
| **RELEVANCE** | | | |
| Is the research question interesting? | Research question is explicitly stated. | The research question is explicitly stated: "This study’s purpose is to use qualitative data analyses of 25 interviews at 18 treatment programs nationwide to identify the challenges alcohol and substance abuse treatment programs for AI/AN communities face in providing meaningful and effective treatment" (lines 108-114). | |
| Is the research question relevant to clinical practice, public health, or policy? | Research question justified and linked to the existing knowledge base (empirical, research, theory, policy) | The "Background" section highlights various factors impacting the quality of substance abuse treatment in American Indian/Alaska Native (AI/AN) communities, including systemic factors and factors unique to AI/AN communities. It sets the stage for the research question (see lines 63-114). | |
| **APPROPRIATENESS OF QUALITATIVE METHOD** | | | |
| Is qualitative methodology the best approach for the study aims?  *Interviews*  *Focus Groups*  *Ethnography*  *Textual Analysis* | Study design described and justified, i.e., why was a particular method (e.g. interviews) chosen? | In the "Methods" section, we describe how we chose to use focus groups of front-line clinical staff from multiple treatment programs to "facilitate richer conversation" (lines 156-158). We also did key informant interview with program staff in clinical administrative positions. Focus group guides, in particular, were designed to "generate open-ended conversation about the community the program serves, the services provided and how they were developed, the challenges of providing these services, and the participants' experience with selected EBTs (evidence-based treatments)" (lines 183-186). Qualitative methodology was appropriate for Phase 2 of the Centers for American Indian and Alaska Native Health’s *Evidence-Based Practices and Substance Abuse Treatment for Native Americans* project. This study’s primary aims are described in in the "Methods" section (see lines 118-125). | |
| **TRANSPARENCY OF PROCEDURES** | | | |
| ***Sampling*** | | | |
| Are the participants selected the most appropriate to provide access to the type of knowledge sought by the study? | Criteria for selecting the study sample justified and explained. | The purpose of this study is to examine the challenges of providing quality substance abuse treatment in AI/AN communities. In the "Settings and Participants" section, we explain how we chose to interview front-line clinical staff in focus groups and key informant interviews with program staff in clinical administrative positions (see lines 149-156). We also note why we chose to interview program staff, rather than patients (see lines 156-161). | |
| Is the sampling strategy appropriate? | *theoretical:* based on preconceived or emergent theory  *purposive:* diversity of opinion  *Volunteer:* feasibility, hard-to-reach groups | The "Settings and Participants" section describes how "all eighteen substance abuse treatment programs invited to participate in the program case study component of this study agreed to participate" (lines 146-148) and that these programs were invited “based on their reputations for innovative clinical services and to assure adequate representation of the geographic, cultural, and reservation/rural/urban diversity of AI/AN communities" (lines 138-140). | |
| ***Recruitment*** | | | |
| Was recruitment conducted using appropriate methods? | Details of how recruitment was conducted and by whom | The "Settings and Participants" section describes how the primary investigator submitted letters of inquiry to program leadership inviting them to participate (see lines 143-146). Programs were assured of confidentiality. All 18 programs agreed to participate. Each individual focus group and key informant participant also completed a formal consent process (lines 180-181). | |
| Is the sampling strategy appropriate? |  | The focus on programs providing innovative services was chosen to understand how highly-perceived programs think about and use evidence-based treatments while being mindful of the unique clinical and cultural contexts for their program. In the discussion section we note that "our focus on programs providing innovative services [may] limit the applicability of these findings to programs that do not meet this criterion" (lines 435-437). | |
| Could there by selection bias? | Details of who chose to participate and why | The "Settings and Participants" section describes how "all eighteen substance abuse treatment programs invited to participate in the program case study component of this study agreed to participate" (lines 146-148). We also note how confidentiality was emphasized to ensure participation and how all 18 programs agreed to participate. The 18 programs were chosen deliberately. Thus, there is clearly selection bias. However, we explain that programs with reputations for innovative clinical services were chosen to fulfill the study's primary aims (see lines 138-140). Furthermore, we provide a table (Table 1, see line 641) that highlights their geographical diversity. | |
| ***Data Collection*** | | | |
| Was collection of data systematic and comprehensive? | Method(s) outlined and examples given (e.g. interview questions) | We provide the website for the focus group and key informant interview guides (see lines 186-188). | |
| Are characteristics of the study group and setting clear? | Study group and setting clearly described. | Yes. See the "Settings and Participants" section (lines 137-175). | |
| Why and when was data collection stopped, and is this reasonable? | End of data collection justified and described. | "Data collection took place from August 2009 through July 2010" (lines 164-165). | |
| ***Role of Researchers*** | | | |
| Is the researcher(s) appropriate? How might they bias (good and bad) the conduct of the study and results? | Do the researchers occupy dual roles (clinician and researcher)? Are the ethics of this discussed? Do the researcher(s) critically examine their own influence on the formulation of the research question, data collection, and interpretation? | The researchers do not occupy dual roles. | |
| ***Ethics*** | | | |
| Was informed consent sought and granted? | Informed consent process explicitly and clearly detailed. | Yes (see lines 180-181). | |
| Was participants' anonymity and confidentiality ensured? | Anonymity and confidentiality discussed. | Anonymity and confidentiality were ensured to both programs and to individuals who participated in the interview (see lines 143-146). | |
| Was approval from an appropriate ethics committee received? | Ethics approval cited. | Ethics approval is cited (see lines 176-181). | |
| **SOUNDNESS OF INTERPRETIVE APPROACH** | | | |
| ***Analysis*** | | | |
| Is the type of analysis appropriate for the type of study? (*thematic:* exploratory, descriptive, hypothesis generating; *framework:* e.g. policy; *constant comparison/ grounded theory:* theory generating, analytical)  Are the interpretations clearly presented and adequately supported by the evidence? | Analytic approach described in depth and justified (*Indicators of quality:*  Description of how themes were derived from data--inductive or deductive; Evidence of  alternative explanations being sought; Analysis and presentation of negative or deviant cases) | The "Data Analysis" section describes how interview transcripts were analyzed using grounded theory to identify key themes and associated subcategories (see lines 204-208). In the results section, we provide our interpretations of the data analyses: "We identified three sets of challenges for bringing effective substance abuse treatment to AI/AN communities: challenges associated with providing clinical support, challenges associated with the infrastructure of the treatment settings, and challenges associated with the service/treatment system. These are summarized in Table 2. Of particular importance was the way these different sets of challenges interact synergistically with one another, creating a highly complex context for the delivery of these services. We first present these three sets of challenges separately for clarity and organization and then discuss their interrelatedness" (lines 210-216). | |
| Are quotes used and are these appropriate and effective? | Description of the basis on which quotes were chosen; Semi-quantification when appropriate; Illumination of context and/or meaning, richly detailed. | Quotes were organized and presented based on the category of challenges they highlighted (see "Results" section). In the final section of the "Results," we present two particularly strong quotations that demonstrate the complex and interrelated nature of the three types of challenges we identified (see lines 348-396).  While we did not quantify our themes, we do provide a table (table 2, lines 643-646) that organizes the themes and subthemes and provides examples. | |
| Was trustworthiness/reliability of the data and interpretations checked? | Method of reliability check described and justified (e.g. was an audit trail, triangulation, or member checking employed? Did an independent analyst review data and contest themes? How were disagreements resolved?). | In the "Data Analyses" section, we note, "Regular discussions among the authors were held to achieve consensus on emerging themes and hypotheses" (lines 207-208). | |
| ***Discussion and presentation*** | | | |
| Are findings sufficiently grounded in a theoretical or conceptual framework?  Is adequate account taken of previous knowledge and how the findings add? | Findings presented with reference to existing theoretical and empirical literature, and how they contribute. | | In the "Discussion" section we describe previously identified challenges to providing substance abuse treatment to AI/AN communities (lines 416-420, lines 424-429). However, we also emphasize the unique nature of our findings, specifically is "the powerful, synergistic interactions between clinical, infrastructural, and service system challenges, which create a highly complex context for the provision of quality substance abuse treatment" (lines 398-400). In terms of our findings' contribution, we note in the "Conclusions" section: "Our framework for conceptualizing these challenges may be useful for exploring these issues other diverse substance abuse treatment settings, enabling clinicians, treatment programs, and policymakers to better assure the provision of the highest quality substance abuse treatment services" (lines 451-454). |
| Are the limitations thoughtfully considered? | Strengths and limitations explicitly described and discussed. | | We present the limitations in the "Discussion" section (see lines 429-437), specifically not including programs serving non-AI/AN communities and focusing on AI/AN substance abuse treatment programs providing innovative services. |
| Is the manuscript well written and accessible? | Evidence of following guidelines (format, word count).  Detail of methods or additional quotes contained in appendix.  Written for a health sciences audience. | | This paper follows the format set forth by *BMC Psychiatry*. The methods section is extensive. The paper is written for a health sciences audience. |
| Are red flags present? These are common features of ill-conceived or poorly executed qualitative studies, are a cause for concern, and must be viewed critically. They might be fatal flaws, or they may result from lack of detail or clarity. | *Grounded theory:* not a simple content analysis but a complex, sociological theory generating approach.  *Jargon:* descriptions that are trite, pat of jargon filled should be viewed skeptically.  *Over interpretation:* interpretation must be grounded in "accounts" and semi-quantified if possible or appropriate.  *Seems anecdotal, self-evident:* may be a superficial analysis, not rooted in conceptual framework or linked to previous knowledge, and lacking depth.  *Consent process thinly discussed:* may not have met ethics requirements.  *Doctor-researcher:* consider the ethical implications for patients and the bias in data collection and interpretation. | | The data was analyzed based upon the principles of Grounded Theory. Jargon is not utilized. Analyses and interpretations of the interview transcripts are not over interpreted, nor are they anecdotal or self-evident. The data analyses were in-depth and linked to previous knowledge. The consent process met ethical requirements, and there are no ethical concerns related to the researchers also functioning simultaneously as physicians. |
